# Saponins in Cancer Treatment: Current Progress and Future Prospects

**DOI:** 10.3390/pathophysiology28020017

**Published:** 2021-06-05

**Authors:** Olusola Olalekan Elekofehinti, Opeyemi Iwaloye, Femi Olawale, Esther Opeyemi Ariyo

**Affiliations:** 1 Bioinformatics and Molecular Biology Unit, Department of Biochemistry, Federal University of Technology Akure, PMB 704, Nigeria; popenapoleon@gmail.com (O.I.); estherariyo87@gmail.com (E.O.A.); 2 Nanogene and Drug Delivery Group, Department of Biochemistry, University of Kwa-Zulu Natal, Durban 4000, South Africa; olawalefemi3@gmail.com; 3 Department of Biochemistry, College of Medicine, University of Lagos, Lagos 101017, Nigeria

**Keywords:** saponins, anticancer activities, traditional plants, mechanism of action, cell-cycle arrest, apoptosis, chemopreventive, future cancer research

## Abstract

Saponins are steroidal or triterpenoid glycoside that is distinguished by the soap-forming nature. Different saponins have been characterized and purified and are gaining attention in cancer chemotherapy. Saponins possess high structural diversity, which is linked to the anticancer activities. Several studies have reported the role of saponins in cancer and the mechanism of actions, including cell-cycle arrest, antioxidant activity, cellular invasion inhibition, induction of apoptosis and autophagy. Despite the extensive research and significant anticancer effects of saponins, there are currently no known FDA-approved saponin-based anticancer drugs. This can be attributed to a number of limitations, including toxicities and drug-likeness properties. Recent studies have explored options such as combination therapy and drug delivery systems to ensure increased efficacy and decreased toxicity in saponin. This review discusses the current knowledge on different saponins, their anticancer activity and mechanisms of action, as well as promising research within the last two decades and recommendations for future studies.

## 1. Introduction

Cancer is a group of diseases that is characterized by uncontrolled cell proliferation. This unconstrained cell growth has the potential to invade nearby and distant tissues causing life-threatening complications [1]. Cancer is a global health challenge and is one of the leading causes of death in both developing and developed countries [2]. An epidemiological study conducted by the World Health Organization (WHO) noted that cancer accounted for the deaths of 7.6 million individuals in 2018, and this figure was expected to double by 2030 [2]. Several treatment options have been sought to treat cancer, the most common of which is chemotherapy. This treatment involves using drugs/chemical agents to destroy rapidly dividing cells and ultimately prevent the spread to other normal cells in the body. Despite the success rate of chemotherapy, patients continue to suffer from several side effects, such as general weakness, fatigue, loss of appetite and infections. In addition, the lack of selectivity and toxicity of Food and Drug Administration (FDA)-approved anticancer drugs has resulted in a significant drawback in the treatment of cancer [3]. Therefore, the search for alternative therapeutic agents in the treatment of cancer is imperative.

Traditional plants contain phytochemical compounds, which are mainly secondary metabolites used by plants to ensure survival and fecundity. Phytochemical compounds of medicinal importance include glucosinolates, alkaloids, triterpenoid, flavonoids, saponins, pigments and tannins. Various studies investigated the use of secondary plant metabolites in traditional medicine. These secondary metabolites displayed different biological activities, such as antimicrobial, anti-inflammatory, cardioprotective, antiviral and anticancer properties. Approximately 60% of anticancer drugs in clinical use and preclinical trials (vinca alkaloids (vinblastine and vincristine)*,* etoposide, paclitaxel, camptothecin, topotecan, irinotecan, curcumin, resveratrol, genistein, allicin, lycopene, diosgenin, beta-carotene, dactinomycin, bleomycin and doxorubicin, paclitaxel and camptothecin) are derived from plants [4,5,6]. These plant-derived anticancer drugs are widely accepted and generally perceived as relatively safe in terms of toxicity. The significant success achieved so far in using natural compounds as chemotherapeutic alternatives has spurred research interest in other secondary metabolites, such as saponins.

Saponins are a class of structurally diverse phytochemicals that are naturally found in higher plants, marine organisms and microorganisms. This group has displayed various pharmacological properties, including anti-inflammatory, antiviral, cardioprotective, immunoregulatory effects and anticancer activity [7,8]. The profound impact of saponins on cancer cells has gained significant research interest in the pharmaceutical sector. These compounds have demonstrated outstanding potential in inhibiting different cancer cells under in vitro and in vivo conditions. Despite the substantial progress made in recent years, the use of saponins as an anticancer agent has faced certain drawbacks, mainly due to their toxicity and poor pharmacokinetic properties. Therefore, this review comprehensively explores the potential of saponins as an anticancer agent by using various mechanisms; this includes the poorly studied pathways, such as those involved in ferroptosis and necroptosis. Furthermore, the current knowledge on the use of saponins as a chemotherapeutic agent and the window of opportunities it presents for future research were also explored.

## 2. Classification of Saponins

### 2.1. Sources of Saponins

Saponins can be obtained from two primary sources, namely natural and synthetic. Saponins acquired from natural organisms are termed “natural”, while those derived from the artificial route via laboratory synthesis are known as “synthetic”.

#### 2.1.1. Synthetic Saponins

Saponins are synthesized artificially by derivatization of saponins obtained from natural sources or via de novo synthesis. Various natural saponins, such as oleanane, ursane, lupane, dammarane, cholestane, spirostane, furostane and cardenolide can be synthesized chemically, using numerous techniques [9]. However, there are some drawbacks to these methods, such as low yield, toxicity and stringent reaction conditions. In recent years, the use of Schmidt trichloroacetimidate in activating sugars has shown great potential [10]. Although the mechanisms involving the chemical synthesis of saponins are beyond the scope of this review, it should be noted that the synthetic approach associated with saponin purification from a natural source forestalls the challenge of low yield and purity [11]. Additionally, this methodology allows for a structure-based optimization that will enable the design of saponins equipped with desirable structural features.

#### 2.1.2. Natural Sources of Saponins

Historically, saponins were primarily derived from vegetables and herbs. Saponins from herbs include soapwort, ginseng, ginsenosides, gypenosides, soapberry rhizomes from the *Liliaceae*, *Dioscoreaceae*, *Agavaceae, Primulaceae*, *Sapotaceae* and *Caryophyllaceae* families [12,13]. Furthermore, different types of saponins can be isolated within the same plant species. Saponins were initially thought to be endemic to plants but were later discovered in non-plant sources. In the last three decades, marine organisms have been identified as significant sources of saponins. More specifically, organisms belonging to the phylum Echinodermata are rich sources of saponins. Tian et al. identified three groups of saponins (asterosaponins, cyclic glycosides and polyhydroxysteroidal glycosides) in starfish and sea cucumbers [14].

### 2.2. Classification Based on the Structure

A typical saponin molecule is made up of distinct structural components consisting of an isoprenoid unit and a sugar residue. The former is referred to as the aglycone component, while the latter is called glycone. Acid hydrolysis of the glycosidic bond between glycone and aglycone of saponins can be used to separate these structural units. The biological activities of saponins are due to their unique structure and amphiphilic nature. It consists of a hydrophilic sugar moiety and a hydrophobic genin (called sapogenin). Additionally, aglycones may possess steroids or triterpenes structure, which is used to classify saponins.

**Figure 1:**
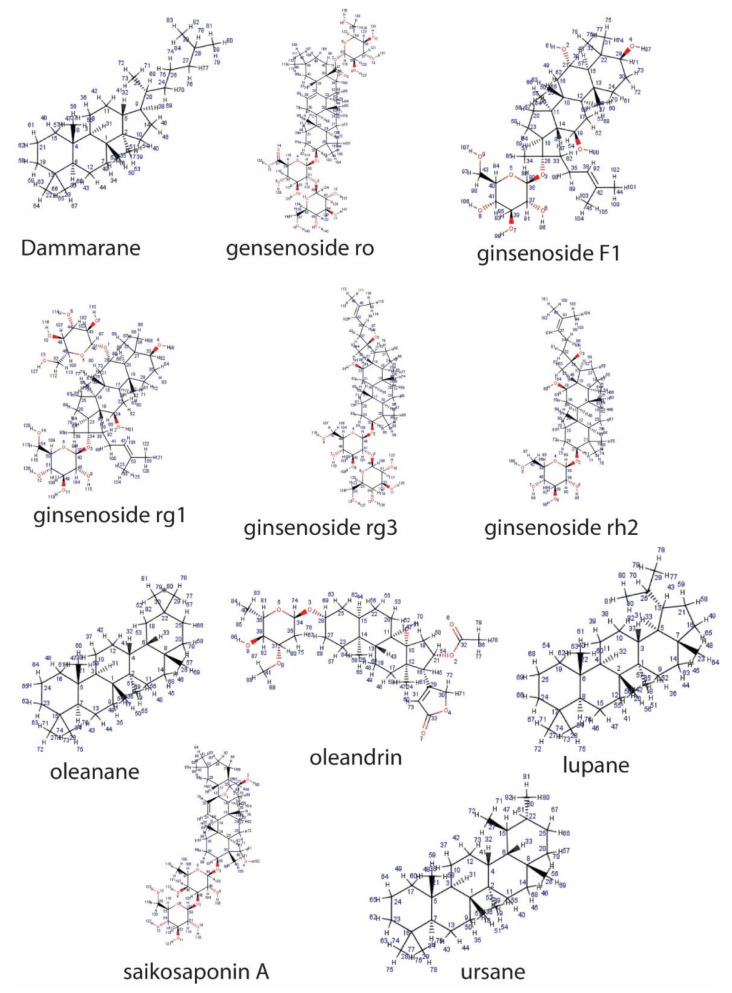
Representative sapogenin structure of triterpenoid saponins.

Triterpenoid saponins (basic) consist of four or five rings, with a 30-carbon backbone structure derived from 2,3-oxidosqualene [15]. The pentacyclic triterpenoids are the most abundant in plants, and they include oleananes, lupanes, ursanes and derivatives (such as saikosaponins) (Figure 1).

**Figure 2:**
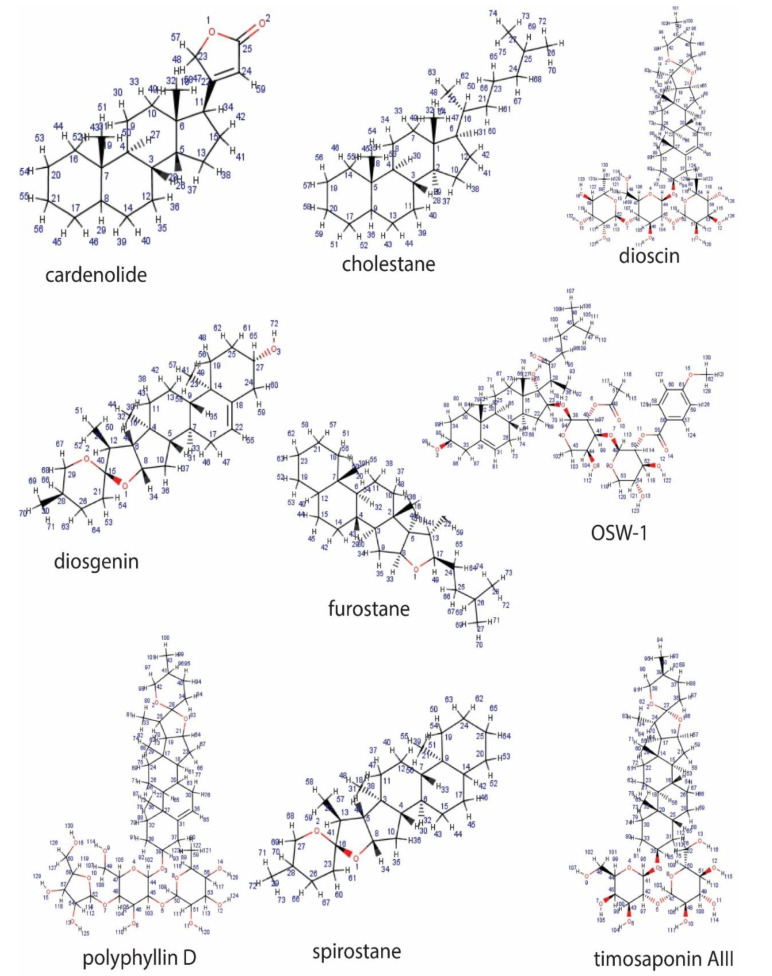
Representative sapogenin structure of steroid saponins.

The less common tetracyclic triterpenoid saponins are dammaranes and their derivatives (including ginsenosides), while the steroidal sapogenins are 27-carbon sugar conjugates of steroids consisting of a five- or six-ring skeleton known as spirostane and furostane, respectively. These include dioscin, diosgenin, polyphyllin D, timosaponin AII, cardenolide and cholestane (Figure 2).

Saponins also differ in structural composition, linkage and the number of sugar chains. Usually, the sugar chain may consist of one or more monosaccharide residues attached at C-3 [16]. Based on the number of sugar residues, saponins are classified as monodesmodic, bidesmodic and polydesmodic, if they contain one, two and more than two sugar residues, respectively. Saponins are also named based on the nature of the sugar residue present on their chain. Glucose containing saponins are regarded as glucosides, while galactose containing saponins are galactosides.

## 3. Anticancer Mechanisms of Saponin

The anticancer activities of saponins include anti-proliferation, anti-metastasis, anti-angiogenesis and reversal of multidrug resistance (MDR). These effects are brought about by induction of apoptosis, promotion of cell differentiation, immune-modulatory effects, bile acid–binding and amelioration of carcinogen-induced cell proliferation [17]. Different molecular mechanisms are involved in the anticancer activity of saponins (Table 1). It should be noted that the mechanism of anticancer action of saponins is strongly related to the nature of the structural moieties, including the aglycone moiety, the length and linkage of the glycosidic chain, the presence of a functional carboxylic group on the aglycone chain, the number of sugar molecules and hydroxyl group, position of the hydroxyl group, stereo-selectivity and the type of sugar molecule on the glycine chain [18,19,20]. This section considers the critical processes in cancer-cell development and how different saponins help to inhibit cancer at various stages.

### 3.1. Chemoprevention and Saponin

**Figure 3:**
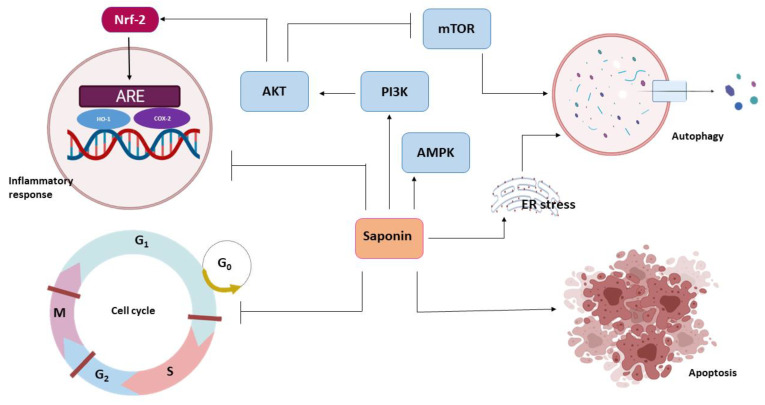
Anticancer effects of saponins.

Chemoprevention is the use of a chemotherapeutic agent to halt or restrict tumor development before the onset of cellular invasion. The chemopreventive action of saponins involves anti-inflammation, redox potential modulation and cell proliferation inhibition through different pathways (Figure 3).

#### 3.1.1. Anti-Inflammatory Activity

The immune system triggers an inflammatory response to foreign invaders as part of the body’s defense mechanism. Nonetheless, excessive or chronic inflammation is associated with different pathological conditions, one of which is cancer [40]. Due to the link between cancer and inflammation, several anti-inflammatory drugs help to decrease the incidence of cancer. Most inflammatory drugs have been designed to selectively target proteins, such as nuclear factor Kappa B (NF-κB), IL-6/STAT3, IL-23/Th-17 and cyclooxygenase-2 (Cox-2), which are responsible for inflammatory response. Similar to other anti-inflammatory drugs, some saponins can regulate the expression of a number of these proteins.

The inducible transcription factor, NF-κB, stimulates the expression of pro-inflammatory and pro-survival genes. These can be activated via a canonical pathway involving TNF-α, T-cell and B-cell receptors. Triggering this protein in cancer cells leads to activation of cell-cycle proteins, metalloproteinase and apoptotic proteins. Reports have identified saponins that inhibit NF-κB and inhibitory kappa B kinase (IKK). For instance, Paris saponin II, a steroidal saponin, inhibits IKK-b, a protein involved in the canonical pathway of NF-κB activation, leading to cell-cycle arrest and apoptosis activation [41]. Moreover, Raddeanin A, a triterpenoid, inactivates NF-κB by preventing the phosphorylation of Ikkb-α. A study by Xia et al. likewise reported the downregulation of the NF-κB signaling pathway by saponins of *Patrinia villosa*, which led to a significant inhibition in colorectal cell proliferation, invasion and metastasis [42].

In addition, saponin fractions from marine spiny brittle starfish extract were found to inhibit TNF-α and Cox-2 [43]. Triterpenoid saponin from *Conyza blinii* showed heightened anticancer activity via p65-dependent NF-κB inhibition [44]. Dammarane triterpenoid isolated from *Cyclocarya paliurus* mediates anti-inflammatory activity by lowering TNF-α, PGE2 and IL-6 expression [45]. Structure-dependent activity studies of different triterpenoid isoforms revealed cyclocarioside X as a potent chemopreventive agent, which shows significant inhibition of COX-2, iNOS (inducible nitric oxide synthase) and NF-κB/p65 in raw 264.7 cells. The role of saponins in regulating proteins involved in inflammatory pathways undermines its critical chemopreventive potentials.

#### 3.1.2. Modulation of Redox Potential

Reactive oxygen species (ROS) encompass free radical oxygen intermediates involved in tumor cell proliferation, genomic instability, resistance to apoptosis and tumor invasion [46]. An imbalance between free-radical production and the antioxidant defense system leads to oxidative stress implicated in cancer initiation. By acting as free radical scavengers, modulating the redox signaling pathway and increasing the expression of antioxidant enzymes, saponins can help to correct the redox imbalance [47]. Purified bacosides, a triterpenoid saponin from *Bacopa monnieri*, has shown significant 2,2-diphenyl-1-picrylhydrazyl (DPPH) radical scavenging activity. Moreover, Choudhry et al. reported that saponin based nano-emulsification improves the antioxidant properties of Vitamins A and E in AML-12 cells [48]. Furthermore, saponins derived from *Panax notoginseng* increase the expression of the antioxidant enzyme heme oxygenase-1 by increasing the phosphorylation of AKT protein and the activity of Nrf2 [49].

Saponins have also shown pro-oxidant activity in cancer cells in addition to their antioxidative activity. Dysregulation of redox signaling is a feature in most cancer cells. Cancer cells survive oxidative burst by upregulating the antioxidant defense system via antioxidant response element (ARE). Blocking cancer-cell antioxidant defense systems would increase ROS-induced oxidative damage, resulting in cancer-cell death [50]. Triterpenoid saponins from *Ardisia gigantifolia* cause cell death in triple-negative breast cancer cells by increasing the generation of reactive oxygen species, activating ERK and AKT and inducing apoptosis via the intrinsic pathway [51]. Kim et al. also observed that hederagenin obtained from *Hedera helix* mediates cell damage in head and neck cancer cells by reducing glutathione reductase activity, increasing ROS and inhibiting the Nrf2-ARE pathway [52].

#### 3.1.3. Cell-Cycle Arrest

Cell progression through the cell cycle is mediated by crucial proteins such as cyclins and cyclin-dependent kinases (CDK) and regulated by checkpoint kinases such as Polo-like kinase, aurora kinase and CDK inhibitors [53]. Cancer cells often show mutations in protein kinases (CDK2, CDK4, CDK6, chk1, Wee1 and PLK1) involved in cell proliferation. Targeting these proteins have become an attractive chemopreventive strategy to mitigate abnormal cell proliferation in cancer cells [54]. Recently, saponins have shown attractive anticancer potentials by modulating cell-cycle proteins, including cyclins, cyclin-dependent kinases and checkpoint proteins, to terminate cancer-cell progression.

Prior to proliferative stimulus, cells in the resting stage (G0) progresses through the G1, S, G2 and M phase of the cell cycle. Different saponins regulate cell progression at each phase of the cell cycle. Furostan-type steroidal genin from edible spears of triguero HT asparagus decreased the expression of cyclin A, D and E by mediating G0/G1 arrest in human colon cancer cells [55]. A similar cell-cycle suppression at the G0/G1 phase has also been observed in Paris saponin VII treated human leukemia cells (K562/ADR) [56]. Saponins also decrease cyclin B1/D1 and CDK2/4/6 protein expression. Chikusetsu saponin IV, a methyl ester of a ginsenoside purified from *Panacis japonica,* has similarly shown the capacity to decrease cell-cycle progression through the S-phase [57]. Moreover, the compound was shown to inhibit the expression of cyclin D1, CDK2 and CDK6. Yaoming et al. reported cell-cycle arrest in the S-phase by triterpenoid saponin from *Camellia sinensis* in the human ovarian cancer cell [58]. The cellular inhibition was achieved by downregulating Cdc25A, Cdk2 and CyclinD1 expression. More so, Paris saponin I have shown G2/M1 arrest in gastric cancer cells by upregulating the activity of p21, a checkpoint protein [59]. Recently, a steroidal saponin purified from the rhizome of *Paris polyphylla* var. *latifolia* was shown to induce the expression of p21 and downregulated the expression of cdc25C, Cyclin B1 and cdc2, thereby inducing G2/M phase arrest in human colorectal cancer [60].

In a normal cell, damage to cellular components (such as DNA damage) will prevent the progress of the cell through the cell cycle. However, cancer cells are unresponsive to proteins associated with the regulation of the cell cycle. Targeting checkpoint proteins such as ChK (checkpoint protein), p21 and Wee1 have become an interesting therapeutic target by many anticancer drugs [53]. Treatment of HepG2 cells with hellebrigenin causes DNA damage activating ATM, Chk1, Chk2 and CDK1/Cyclin B1 kinase resulting in G2/M-phase cell-cycle arrest [61]. Diosgenin has similarly shown activation of Cdc25C phosphatase, which triggers the Cdc2-cyclin B pathway mediating G2/M cell-cycle arrest in breast cancer [62].

### 3.2. Cytotoxicity Effects of Saponins

In addition to side their chemopreventive actions, saponins show cytotoxic effects in cancer cells. Saponin treatment in cancer cells can stimulate autophagic cell death, decrease nitric oxide production and cause cytoskeleton integrity disassembly. Their cytotoxic effects can be initiated either by apoptosis or non-apoptotic stimulation of cell death. Extensive literature search has revealed the significant ability of saponins to induce cancer-cell death through apoptosis, ferroptosis, oncotic necrosis, necroptosis and autophagy.

#### 3.2.1. Apoptosis

Apoptosis is a programmed form of cell death characterized by cell shrinkage, chromatin condensation, nuclear fragmentation and membrane blebbing. It may be initiated either at the plasma membrane (extrinsic pathway) or inside the cell and is critical in regulating tissue development and homeostasis [63]. Apoptosis is the most studied form of cell death, and unlike other forms of cell death, it is well regulated and not accompanied by an inflammatory response. The induction of apoptosis of tumor cells is an effective way of treating tumours. Compelling evidence has shown that most cytotoxic agents used in cancer therapy can induce apoptosis [63].

**Figure 4:**
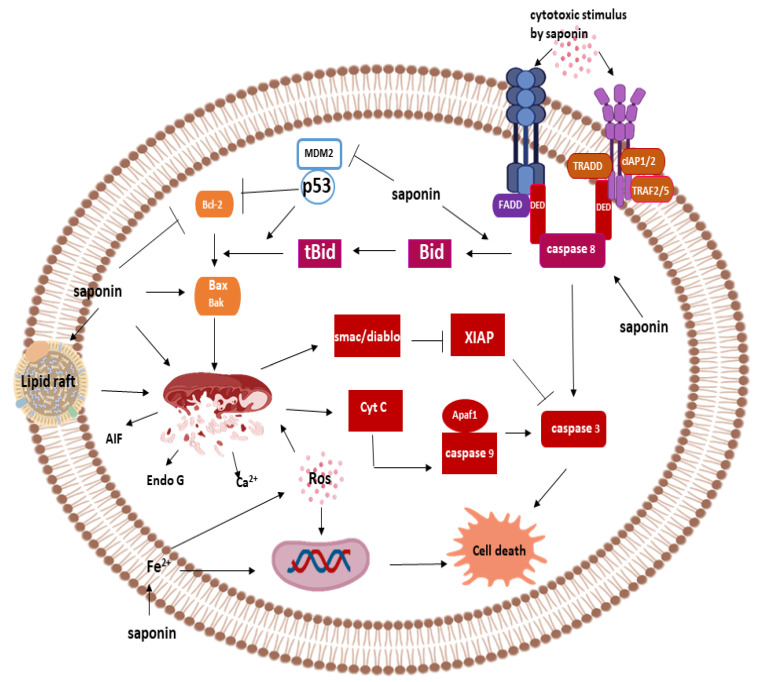
Cell-death mechanisms of saponins.

Saponins can induce apoptosis through a series of reactions involving the activation of a protease family of enzymes known as caspase. Other caspases independent apoptosis pathways have also been described in the mechanism of cell death by saponins. In this section, we consider the cellular mechanism of cell death by saponins and elucidate the underlying molecular mechanism of the induction (Figure 4).

##### Saponins and Caspase-Dependent Apoptosis

Caspases are cysteine dependent aspartate specific proteases that mediate the initiation and execution phase of apoptosis. These enzymes are synthesized in their inactive form known as pro-enzyme or zymogens and can be activated via a receptor-mediated pathway or the mitochondria-dependent pathway. While the former is known as the extrinsic pathway, the latter also called the intrinsic pathway. Saponins can initiate a caspase-dependent pathway of apoptosis via both the extrinsic and intrinsic pathway.

##### Extrinsic Pathway and Saponins

The extrinsic pathway is initiated by the binding of ligand to members of the TNF superfamily of protein, including Fas receptor, TNF-α and TRAIL. Saponins can activate the extrinsic pathway of apoptosis by activating the Fas receptor leading to the recruitment of adaptor molecule called Fas-associated death domain (FADD) [64]. The recruitment of FADD triggers the conscription of Pro-caspase 8 in saponin treated cancer cells to form death induced signaling complex (DISC) [63,65]. Upon recruitment, procaspase-8 is released from DISC as the active caspase-8 via a proximity-induced activation mechanism. Activation of caspase-8 leads to downstream activation of the executioner caspase-3 and cleavage of poly-ADP-ribose polymerase (PARP) mediating the proteolysis of cellular components [66]. Caspase-8 activation is the defining factor in the extrinsic apoptosis pathway, and activation of this protein can be induced by different saponins [67].

Cellular activation of caspase-8 by saponins via the intrinsic pathway might not be sufficient to induce apoptosis [64]; as a result, some saponins rely on cell death machinery via the BCl-2 family of protein (Bid) which mediates crosstalk with the intrinsic apoptosis pathway [68]. Caspase-8 activates tBid by protein cleavage to form an active Bid, which subsequently activates downstream pro-apoptotic proteins, Bax and Bak, causing mitochondria membrane permeabilization and activation of effector caspases [69]. Furthermore, since p53 mutation in cancer cells can inhibit apoptosis in the intrinsic pathway, this pathway of cell death offers an alternative route of eliminating cancer in p53 mutant cells [69].

##### Intrinsic Pathway and Saponins

The intrinsic pathway is a mitochondria-dependent pathway of apoptosis, and it is the most reported mechanism of apoptosis induction by chemotherapeutic agents. Saponins can stimulate the release of pro-apoptotic factors, cytochrome C, Ca^2+^ and Smac/DIABLO, from the mitochondria via cytotoxic action or ROS production [70,71]. These ROS/cytotoxic stimuli disrupt the mitochondria to initiate the apoptosis process. Pro-apoptotic cytochrome C binds Apaf-1 to form the apoptosome complex required for the activation of pro-caspase-9. Upon activation, caspase-9 cleaves executioner caspase-3, activating the protein and the downstream apoptosis process. Activation of apoptosis by saponins via the intrinsic route involves the inhibition of anti-apoptotic protein Bcl-2 and activation of pro-apoptotic proteins caspase-9 and caspase-3 [72].

Saponins also mediate the intrinsic pathway via mechanisms involving the activation of p53 proteins [73]. Activation of p53 can be achieved by the inhibition of MDM2 via direct interaction or by binding to the alternative reading frame (ARF) [74]. Activation of p53 causes the inhibition of anti-apoptotic Bcl-2 and activation of pro-apoptotic Bax, Noxa and Bad, leading to depolarization of mitochondria and the release of cytochrome C from the mitochondria [64]. This protein then mediates executioner caspase -3 and -9 activation [65]. Furthermore, saponins can stimulate Smac/Diablo to subsequently inhibits the activity of XIAP (inhibitor of executioner caspase-3), thereby stimulating apoptosis [67].

##### Saponin and Caspase Independent Apoptosis

Saponins are capable of inducing cell death via pathways independent of caspases but show morphological features typical of apoptotic cell death. In this form of cell death, caspases are not activated, and their stimulation may not play any active roles in mediating cell death [75]. Before the permeabilization of the mitochondria, different pro-apoptotic factors are released into the inter-membrane space, some of which are cytochrome C, Ca^2+^, Smac/DIABLO, HtrA2/Omi, AIF (Apoptosis-inducing factor) and Endonuclease G. While some of the proteins mediate apoptosis via the intrinsic pathway as earlier discussed, AIF, HtrA2/Omi and Endo G translocate into the nucleus where they bind to DNA resulting in chromatin condensation.

Preceding the release of pro-apoptotic proteins (such as AIF) is the permeabilization of the membrane—this process plays a critical role in the overall apoptosis process and is termed as the committed steps. One of the alternative pathways of cell death induction by saponins involve pore formation on the membrane [76]. Saponins are capable of binding to the cholesterol-rich segment of the membrane or the membrane lipid raft. The cytotoxicity of some saponins can be greatly influenced by the cholesterol content [77]. The binding of saponins to the lipid raft may be the initial upstream process of mediating cytotoxic activity in multidrug-resistant cancer cells before the release of pro-apoptotic Endo-G and AIF [78].

AIF and Endo G have become attractive targets due to their role in caspase-independent apoptosis. Saponins, including dioscin, can induce caspase-independent apoptosis by activating AIF [25]. Although the activation of AIF by saponins is linked to increased ROS generation, a ROS-independent mechanism activation has also been described [25,79,80,81]. Also, saponins can stimulate the release of Endo G, resulting in their migration to the nucleus, where these bind to chromatin and break the phosphodiester linkage in the nucleotide chain to generate nucleosomal fragments [82,83]. In addition to the pivotal role played by Endo G and AIF, HtrA2/Omi also mediates caspase-independent apoptosis. This pathway of death mechanism may prove an invaluable tool to destroy cancer cells resistant to caspase activation.

#### 3.2.2. Ferroptosis, Oncotic Necrosis and Necroptosis

Ferroptosis is an iron-dependent programmed cell death characterized by the accumulation of lipid peroxides [84]. Different saponins such as ardisiacrispin B, spirostanol saponin, diosgenin saponin, oleanane triterpenoid saponin derivatives and ruscogenin have demonstrated iron-dependent programmed cell death following treatment on cancer cells [85,86,87]. Cancer cell depends on iron for DNA synthesis—an essential step in the cell cycle. Iron overload, however, can cause oxidative damage in cancer cells via the Fenton reaction [84]. This mechanism holds great promise to prevent both drug-sensitive and resistant cancer cells from proliferating [87].

Furthermore, Gao et al. discovered a novel form of cell death in tumor cells in which exposure to trisaccharide saponin derivatives induced cell swelling followed by cell membrane perturbation and destruction of the cytoskeletal network in the form of cell death known as oncotic necrosis [88]. Polyphyllin D and progenin III can induce programmed necrosis/necroptosis in cancer cells [26,89,90]. The molecular mechanism by which saponins exert necroptosis is not fully known, but similar to apoptotic cell death, it involves the activation of Caspase 8 as observed in the extrinsic pathway of apoptosis [26].

#### 3.2.3. Autophagy and Saponin

Autophagy is a mechanism adopted by cells to remove dysfunctional or redundant cellular components, which are later recycled to meet the metabolic needs of starving cells. It plays a dichotomous role in cancer-cell death and pro-survival mechanisms [91]. Autophagy can cause apoptotic cell death, but it may also help cancer cells survive oxidative stress and metabolic stress by recycling defective cell components. Despite the significant progress made to understand the mechanism of autophagy, the question of whether to stimulate or inhibit autophagy in cancer therapeutics remains debatable [92]. Several studies on cancer have shown that autophagy promotes cell survival in cancer cells; however, excessive autophagy exceeding cellular repair capacity stimulates cell death [93]. While most anticancer agents seem to inhibit autophagy, some have also shown an ability to stimulate autophagy. Purified *Pulsatilla* saponin D (SB365) from *Pulsatilla chinensis* showed a dual role by inducing the early event of autophagy (autophagosome formation) and inhibiting the latter stage of autophagy (autophagic flux) [94]. Zhang et al. noted that SB365 increased microtubule-associated protein 1A/1B-light chain 3 (LC3) and p62 expressions in HeLa cells [94]. The LC3 protein is involved in the formation of autophagosome, while p62 can degrade LC3 protein to inhibit autophagic flux. The authors, however, concluded that the inhibition of autophagic flux by increasing p62 expression might play a significant role in the anticancer activity of SB365 against HeLa cells. 

Different molecular pathways, including mTOR, MAPK, AMPK and JNK, are implicated in the regulation of autophagy [95]. However, the PI3/Akt/mTOR signaling pathway, which mediates crosstalk between autophagy and apoptosis, appears to be the most studied [93,96]. Xie et al. reported the induction of autophagy by Paris saponins from *Paris polyphyllae* through the downregulation of Akt/mTOR in breast cancer cells [97]. Triterpenoid glycosides are also reported to induce apoptosis in hepatocellular carcinoma by modulating the PI3K/Akt/mTOR signaling pathway [98]. Promoting autophagy through mTOR inhibition might be an effective way of cancer chemoprevention by preventing the accumulation of metabolic stress [93,99].

The cytotoxic stress response is another mechanism through which autophagy can be activated. This process involves the P13/AKT pathway and can be stimulated by saponin [100]. The saponins extracted from *Camellia sinensis* flowers induced ROS dependent autophagy in ovarian cancer cells, resulting in the activation of the MAPK signaling pathway [101]. Recent evidence has suggested that a specific protein known as AMPK can act downstream of MAPK to induce autophagy [102]. The AMPK protein is an energy stress response protein that facilitates metabolic activity in cells to generate more ATP, which in turn causes oxidative stress through the generation of ROS. In NSCLC cells, treatment with Paris saponin VII was shown to increase the expression of AMPK and its downstream effector, ulk1, which are critical in inducing autophagy [103]. In vitro and in vivo studies have also demonstrated the induction of autophagy by saponins via the JNK pathway [104,105].

Due to autophagic flux often associated with the growth of tumours, recent studies have primarily focused on identifying autophagy inhibitors [91,92,106]. Moreover, inhibiting autophagy help in preventing tumor immune invasion [92]. A study by Liu et al. identified triterpenoid saponins from *Conyza blini* capable of eliciting cytotoxic activity in HeLa cells through the inhibition of autophagy [107]. Paradoxically, activation of autophagy has been shown to trigger T-cell cytotoxicity reducing cancer-cell growth [108]. Therefore, further studies are needed to understand how saponins modulate autophagy to prevent cancer progression.

### 3.3. Metastasis and Saponins

In chronic cases, tumor cells migrate from their primary site through the lymphatic or blood system and subsequently colonize distant tissues and organs [109]. This process is known as tumor metastasis, and it accounts for 90% of cancer-associated mortality [110]. Metastatic cancer cells have acquired multiple genetic alterations that enable them to survive at a distant site. The processes involved in metastasis are quite complex because these entail different alterations that result in stimulation of angiogenesis, local invasion attachment, basement membrane disruption, matrix proteolysis and stimulation of growth factors among others [111].

Angiogenesis is the formation of new blood vessels from pre-existing vessels to deliver nutrients and oxygen to a distant site, and it is critical for the colonisation of secondary tumours. Saponins have been identified with the potential to inhibit the formation of new blood vessels in tumor cells [112]. For example, ginsenoside-Rb2, a dammarane saponin, slows down tumor metastasis of B16-BL6 by inhibiting tumor-induced angiogenesis [113]. Chan et al. highlighted that polyphyllin D suppresses the proliferation and migration of endothelial cells in vitro and inhibits intersegmental vessel (ISV) formation in zebrafish [114]. Similarly, *Panax notoginseng* has also been shown to restore defective ISV in zebrafish larva [115].

Yang et al. also reported that *Paris saponin* II (PSII) inhibited angiogenesis at low concentration in cancer cells and showed no toxicity to normal endothelial cells [116]. The anti-angiogenic activity was linked to the potential of PSII to modulate the expression of NF-κB. By downregulating NF-κB expression, PSII reduced the activity of the downstream proteins such as VEGF, Bcl-2 and Bcl-xL. The VEGF protein has been implicated in angiogenesis and lymphogenesis, and its activity is mediated by binding VEGF receptor (a tyrosine kinase receptor). Raddeanin A (RA) is an active triterpenoid saponin from the traditional Chinese medicinal herb *Anemone raddeana* which inhibits the phosphorylation of VEGFR2 by VEGF [117]. The authors noted that RA binds to the ATP binding pocket of VEGFR2 and hinders its phosphorylation by VEGF, thereby preventing the activation of downstream effector proteins such as PLCγ1, JAK2, FAK, Src and Akt [117]. Additionally, sulfated saponin purified from sea cucumber can inhibit the phosphorylated form of VEGFR2 and the consequent downstream signaling pathway required for the mitogenic activity of VEGF in the endothelial cell [118]. 

Another mechanism through which saponin interferes with metastasis is by inhibiting cell adhesion molecules. Attachment of tumor cell to extracellular matrix (EM) and other similar cells is important for metastasis. Different proteins such as integrins, CD44, ICAM and VLA-4 are responsible for the cellular attachment of cancer cells [111]. *Paris polyphylla* can decrease the expression of intracellular adhesion molecule-1 (ICAM-1) in cancer cells [119]. Furthermore, Wang et al. have also reported significant inhibition of inflammation-induced endothelial adhesion molecule by saponin from *Panax notogingseng* [120]. Likewise, a saponin monomer from dwarf lilyturf tuber inhibits hypoxia-induced integrin expression in the human breast cancer cell [121].

Cancer cells undergoing metastasis show a lack of adhesion by inhibiting molecules such as E-cadherin required for homotypic cell–cell interaction. Reduced expression of the adhesion molecule E-cadherin in cancer cells increases cell mobility, as such molecules that increase E-cadherin expression impede tumor metastasis. The activity of E-cadherin is regulated by protein such as Cdc42 and Rac1 [109]. These proteins are Rho GTPases, and their expressions are upregulated by saponins. For example, saponin fractions from *Asparagus officinalis* activate Cdc42 and Rac1 [109]. Furthermore, Ardipusilloside I also stimulate the activity of Rac1. By stimulating these upstream proteins, saponins can inhibit cell migration [122].

Perhaps one of the most studied deregulations in metastasis is tissue remodeling. It involves a family of proteins known as the matrix metalloproteinase (MMP). During metastasis, the tumor cell traverses the extracellular matrix (EM) barrier. This process is critical for cancer-cell invasion, and it includes the proteolytic degradation of the EM by enzymes such as MMP2 and MMP9. Upregulation of MMP-2 and MMP-9 are particularly noted in cancer cells. By targeting multiple proteins participating in tissue remodeling pathways, saponins can significantly reduce cancer metastasis under in vitro and in vivo conditions [112,123]. Several saponins have been identified with significant potential to specifically inhibit matrix degeneration protein such as MMP-2, vimentin and MMP-9 [123,124]. In particular, ginsenoside Rd inhibits the expression of MMP-2, MMP-1 and MMP-7 [125].

Furthermore, NF-κB and certain protein kinase (such as MAPK, ERK, JNK, p38 and P13/AKT) regulate epithelial-mesenchymal transition (EMT) proteins (MMP and MMP2). [126]. The inhibitory potential of specific saponins is linked to their ability to suppress the phosphorylation of some of these protein kinases and inhibit TNF-α induced NF-κB activation [42,127]. For example, kalopanaxasaponin A, a triterpenoid saponin, inhibits the expression of MMP-9 in breast cancer cell by modulating P13/AKT and PKC pathways [128]. Ginseng saponin also inhibits MMP-9 in human astroglioma cell expression by suppressing activator protein-1 and MAPK [129]. In addition, trillium saponins downregulate MMP-2 and MMP-9 expression in HuH-7 cells [130].

The activity of matrix metalloproteinase can be further regulated by endogenous inhibitors, including tissue inhibitors of metalloproteinase (TIMP) and extracellular inducers of matrix metalloproteinase (EMMPRIN) [124,131]. While the former works by reducing the activity of MMP, the latter stimulates the activity of MMP. Diosgenin, a steroidal saponin, inhibits EMMPRIN and stimulates TIMP-2 expression in PC-3 cells [132]. Moreover, soybean saponins can stimulate TIMP-2 expression in colon cancer cells [131]. Similarly, Shuli et al. reported the upregulation of tumour cell TIMP-2 expression following *Rhizoma paridis* saponin treatment [127]. However, further studies are needed to understand MMP role in cancer and their regulation by saponins since it has been observed that increased TIMP-2 expression in glioblastoma patients is accompanied by severe adverse effects [133].

At the secondary site, tumor cells rapidly proliferate as a result of increased levels of growth factors. Different autocrine and paracrine growth factors such as bFGF, IGF-I and EGF are released by metastatic cells [111]. These growth factors have become therapeutic targets for certain anticancer drugs since their stimulation is essential for the rapid growth of cancer cell at distant sites [134]. Saponin DT13 can potentially block metastasis through the inhibition of tissue factor (TF) [121]. Timosaponin AIII suppresses hepatocyte growth factor-induced tumor invasion [36]. Zhuang et al. also reported that dihydrodiosgenin inhibited metastasis by suppressing endothelial cell-derived factor VIII and altering platelet function [135]. Beyond the direct role of the saponins on multiple pathways involved in metastasis, saponins can also be broken down in the body to yield secondary metabolites with potential anti-metastasis activity. For example, saponin metabolites from gut metabolism have shown significant metastasis inhibitory activity [136]. 

### 3.4. Saponin in Multidrug Resistant Cancer

The difficulty associated with drug resistance remains a hurdle in the chemotherapeutic treatment of cancers. Several reports have documented the anticancer activity of saponins against drug-sensitive and drug-resistant cancer-cell lines. Drug resistance in cancer is linked to several determinants, including increased tumor burden and metastasis, multiple chromosomal aberrations, physical barriers to chemotherapeutic agents, tumor micro-environment, adaptive cancer immune response and untargeted oncogenic drivers [137]. Saponins have been described as being able to modulate some of the target effectors, such as pgp (p-glycoprotein) and Ras, to elicit cytotoxic activity against resistant cancer cells. Saponins have shown potent p-glycoprotein (an efflux pump highly expressed in many cancer resistant cells) inhibiting activity [138,139]. Similarly, in resistant cell lines, the Ras protein (an oncogenic driver) can be inhibited by Paris saponin VII to stop colorectal cancer from spreading [140]. Some saponins have also demonstrated the ability to reverse multidrug resistance in cancer cells and target angiogenesis in resistant cell lines [123,139]. As a result of their potency to treat multidrug resistance, saponins are explored in combination therapy with other standard drugs to increase the therapeutic effect of current anticancer regimens against drug-resistant cancer cells [141].

Another challenge in cancer therapy is the elimination of cancer stem cells. These cells are capable of growing after effective treatment with chemotherapeutic agents [142]. Interestingly, saponins have shown inhibitory activity against cancer stem cell via a cell death mechanism involving the Wnt/β- catenin signaling pathway [22]. 

## 4. Limitations and Prospects

The number of purified saponins with anticancer activity has increased significantly over the last two decades. Despite the widespread research and reports on the anticancer property of saponins and their derivatives, there are no FDA approved saponin-based anticancer drug [143]. Most of the studies describing the anticancer effect of saponins are from in vitro experiments, and there are only limited in vivo and clinical trial data currently available. This limitation is a result of many factors ranging from drug-likeness property to toxicity index. There have also been concerns about the purity of natural saponins and their availability. This section considers the factors limiting the success of saponins as anticancer drugs and the future directions for better outcomes.

Saponins possess a significantly high molecular weight (around 741 to 1808 Da) and a consequent high number of rotatable hydrogen bonds, total polar surface area and hydrogen bond donors and acceptors [143]. Generally, drugs with low molecular weight, high lipophilicity and fewer hydrogen bond donors and acceptor are usually more bioavailable [144]. Saponin glycone has a notably lower oral bioavailability compared to aglycone saponin [145]. For several low orally bioavailable drugs, high dose oral administration or alternative route such as intravenous and intramuscular routes are usually explored. However, intravenous administration of saponins is not a likely option to be explored since studies have shown that saponins possess high hemolytic activity, which may lead to anemia [143]. The hemolytic activities of saponins are mediated by erythrocyte membrane permeabilization via interaction with the cholesterol of the plasma membrane [146,147]. This activity is linked to critical carboxylic and hydroxyl functional groups of triterpenoid saponins [148].

The activity of saponins is dose-dependent, and a significant increase in oral dosage would mean a significant increase in bioavailability and action, which can significantly increase saponin toxicity [144]. Sub-acute, acute and chronic dose of saponins is associated with nephrotoxicity, hepatoxicity and cardiotoxicity [149,150]. There are, however, reports of non-toxic saponins even at higher concentration following oral administration in animal models [151,152]. While several saponins are hemolytic, a few recently identified saponins, including soya sapogenol, *Astragalus membranaceus* saponins and *Bupleurum chinense* saponins, have been shown to be non-hemolytic [153,154].

Structural optimization of saponin may prove to be very important in improving the drug-like property of saponins. Several anticancer drugs obtained from plants, such as paclitaxel, are structural derivatives of plant compounds. A detailed understanding of the structure-activity relationship of saponins would prove as an invaluable tool to guide the development of bioavailable saponins as a potential anticancer drug candidate. Studies on QSAR of saponins to identify the functional groups responsible for the hemolytic and cytotoxic activity have shown promising outcomes, in addition to structural modification to ensure selective action of the saponins. For example, QSAR and QSPR studies of saponins isolated from *Pulsatilla chinensis* showed that cytotoxic activity of the saponin was independent of its hemolytic activity. This technique would help to identify potent cancer-specific drug candidates [146].

Targeted drug delivery is an alternative approach that could be explored further to increase the efficacy of saponins. Nanoparticles, due to their size, can evade clearance by plasma binding protein and reticuloendothelial system. Nano-encapsulation not only extends the drug circulation time, but it also reduces the toxicity to normal cells. For instance, loading saponins into human serum albumin nanocomposites resulted in improved anticancer drug efficacy and no toxicity to healthy cells [155]. In addition, drug-delivery vehicles involving micelles, self-assembled nano drugs and liposomes can be functionalized by targeting moiety, such as cell-penetrating peptides, to improve selectivity and reduce toxicity [156,157].

One of the most promising areas in saponin anticancer research is combination therapy. There is different evidence that has shown that saponins can be combined with other chemotherapeutic and radiotherapy treatments to improve efficacy and reduce toxicity [32,158]. In combination with radiation treatment, saponins induce apoptosis and cell-cycle arrest in cancer cells, thereby sensitizing resistant cancer cells to radiation treatment [159,160]. Similarly, Saponins are also utilized as adjuvants to boost the body’s immunological response against cancer [141,161]. In addition, saponins have also been shown to have synergistic therapeutic effects when combined with conventional anticancer drugs [80,162]. Targeted saponin delivery and combination therapy appear to hold the best promise for developing saponin derived anticancer agents in the near future.

## 5. Concluding Remarks

The overwhelming evidence from several studies has shown the different anticancer effects of saponins. Previous research has largely linked the anticancer action to membrane permeabilization (which leads to apoptosis); however, more recently discovered saponins have demonstrated enhanced chemopreventive and chemotherapeutic action, utilizing different cytotoxic pathways. Some of these saponins have been demonstrated to have antioxidant properties as well as the ability to control the expression of proteins involved in cell cycle, cancer progression and metastasis. Despite the progress made so far in the use of saponin for cancer treatment, toxicity and low bioavailability remain significant obstacles. Moreover, another difficulty is the fact that the role of diverse saponin scaffolds in anticancer action is unknown, making drug optimization challenging. Combination therapy and more efficient drug delivery technologies, both of which have been used in saponins research have shown the best promise so far. The evidence from these studies, on the other hand, is primarily from in vitro investigations and is quite limited. Further structure-dependent activity and preclinical and clinical studies are therefore essential to ensure the translation of saponin based anticancer drugs from bench to bedside.

## Figures and Tables

**Table 1:** Anticancer activities of saponins and sapogenins.

Compound	Cells/Tissue Type	Molecular Target	References
Diosgenin	MCF-7, breast cancer	The activation of p53, disruption of intracellular Ca^2+^ homeostasis, generation of ROS and caspase activation	[21,22]
Dioscin	Leukemia, lung cancer, gastric carcinoma, hepatocellular carcinoma, cervical cancer, breast cancer	Upregulates FADD, p53, Bid and Bax. Downregulates CDK2,Bcl-2, Clap-1 and Mcl-1	[23,24,25]
Polyphyllin D	Ovarian cancer, cervical cancer, breast cancer, glioblastoma, glioma	Upregulates p53, p21, PDI and JNX. Downregulates CDK1, Bcl-2, HIF- and VEGF	[26,27,28,29]
Oleandrin	Pancreatic cancer, prostate cancer, breast cancer, lymphoma, melanoma, osteosarcoma	Upregulates Akt, ERK and ROS. Downregulates NF-κB, MAPK, JNK, pS6, p4EPB1, PI3K/Akt and mTOR.	[30]
Ginsenoside Rg3	Lung cancer, esophageal carcinoma, gastric cancer, colon cancer, hepatoma, renal cancer, bladder cancer, breast cancer, ovarian cancer, prostate cancer and melanoma	Upregulates p63,p21, Bax and Smac Downregulates VEGF, p38 and P13K,	[17]
Ginsenoside Rh2	Leukemia, colon cancer, hepatocellular carcinoma, breast cancer, ovarian cancer, prostate cancer	Upregulates p53, p21, p27 and p16 Downregulates AKT, CDK4, CDK6 and AP-1.	[17]
Saikosaponin A	Hepatocellular carcinoma, breast cancer, colon cancer	Upregulates p15, p16, ERK and cleaved-PARP Downregulates Bcl-2, XIAP, Clap2 and Pgp	[31]
Saikosaponin D	Lung cancer, hepatocellular carcinoma, prostate cancer, thyroid cancer	Upregulates p53, p21, Fas and Bax, Downregulates Bcl-2, CDK2, COX-2 and STAT3	[32]
Polyphyllin D	Human non-small-cell lung cancer NCI-H460 cell line.	ER stress-mediated apoptosis, induction of tumor suppressor p53, disruption of mitochondrial membrane and activation of caspase-9 and caspase-3	[33]
Timosaponin AIII (TAIII)	Breast, prostate, HepG2, pancreatic and osteosarcoma cancer cells. PANC-1 cell xenograft nude mice model	ER stress induction, activation of caspase-3, downregulation of Bcl-2, X-linked inhibitor of apoptosis protein (XIAP), Mcl-1 and IAPs, induction of cytochrome c and stimulation of caspases 3, 7, 8 and 9	[34,35,36]
OSW-1(3β,16β,17α-trihydroxycholest-5-en-22-one16- O -(2- O -4-methoxybenzoyl-β- D -xylopyranosyl)-(1→3)-(2- O -acetyl-α- L -arabinopyranoside)	Leukemia cancer and pancreatic cancer cells	Mitochondria membrane permeabilization. Intrinsic apoptosis. Calcium-dependent GRP78 (survival factor) cleavage. Binding to oxysterol binding protein to activate the Golgi stress response leading to apoptosis	[37,38,39]

## Data Availability

Not applicable.
